# Muscle morphometric effect of anterior cruciate ligament injury measured by computed tomography: aspects on using non-injured leg as control

**DOI:** 10.1186/1471-2474-14-150

**Published:** 2013-04-29

**Authors:** Sören Strandberg, Maria Lindström, Marie-Louise Wretling, Peter Aspelin, Adel Shalabi

**Affiliations:** 1 Department of Clinical Science, Intervention and Technology at Karolinska Institutet, Division of Medical Imaging and Technology, Stockholm, Sweden and Department of Radiology, Karolinska University Hospital in Huddinge, Stockholm SE-14186, Sweden; 2 Department of Clinical Science, Intervention and Technology at Karolinska Institutet, Division of Orthopedics, Stockholm, Sweden; 3 Department of Clinical Science, Intervention and Technology at Karolinska Institutet, Division of Medical Imaging and Technology, Stockholm and Centre for Medical Imaging, Akademiska Hospital, Uppsala, Sweden

**Keywords:** Anterior cruciate ligament, Muscle size, Atrophy, Dominance, Gender, Meniscal tear, Computed tomography

## Abstract

**Background:**

Anterior cruciate ligament (ACL) tears are common, functionally disabling, and predispose to subsequent injuries and early onset of osteoarthritis in the knee. Injuries result in muscular atrophy and impaired muscular activation. To optimize surgical methods and rehabilitation strategies, knowledge of the effects of ACL injuries on muscles size and function is needed. Asymmetry due to limb dominance implies that the effect of ACL-injury might be different in right-sided and left-sided injuries which, should be taken in account when evaluating the effect of an injury. Evaluation of the effects of injuries is usually made with the contralateral leg as control. The aim of this study is to describe the effect of ACL-injuries on thigh muscle size and also to analyze feasibility of using contralateral limb as control.

**Methods:**

Sixty-two patients scheduled to undergo ACL reconstruction were examined with computed tomography (CT). Muscle cross sectional area (CSA) was recorded for quadriceps, hamstrings, gracilis and sartorius 15 cm above the knee joint. Comparisons were made between the injured and non-injured side and between individuals separated by gender and side of injury. Comparisons were also made for patients with or without concomitant meniscal tear, for patients differing in time between injury and examinations and for patients with different level of physical activity after the injury.

**Results:**

Quadriceps CSA was 5% smaller on the injured side. There was an indication that the muscles of the right thigh were generally bigger than those of the left thigh. The difference between the injured and the non-injured side was larger for right-sided injuries than for left-sided. There was also a greater difference in semimembranosus for women than for men. There were no differences related to meniscal injury, time since injury or physical activity.

**Conclusion:**

The use of contralateral leg for evaluating the effect of ACL-injury is often the only available alternative but our study indicates that the difference in CSA between injured and non-injured side does not necessarily reflect the true degree of atrophy, as there are side differences both in muscle size in general and in the effect of an ACL-injury on muscle size.

## Background

Anterior cruciate ligament (ACL) tears are common among younger persons involved in different sport activities. These injuries are functionally disabling and predispose to subsequent injuries and of osteoarthritis in the knee. The ACL-deficient knee is often characterized by decreased muscle strength and torque generating capabilities, which may be attributed to muscle atrophy [1-4] and impaired muscular activation [4-8], the latter discussed in a review article by Ingersoll et al. 2008 [9]. Beynnon et al. published in 2005 [10] a review article on biomechanical background, prevalence, risk factors, outcome and indications for various treatment regiments as well as on different surgical techniques and rehabilitation strategies [11]. The rationale for and against reconstructive surgery has recently been outlined by Hurd et al. [12]. It is therefore of importance to assess the morphological effect of ACL-injuries in order to develop the best strategies for rehabilitation. ACL-injuries are often accompanied by meniscal tear, which also are known to induce atrophy of quadriceps [13].

### Contralateral leg for comparison: lateral asymmetry and dominance

Most studies use the contralateral limb as comparison in evaluating changes in muscle size and strength following ACL rupture. The underlying assumption that both limbs were equal in size and muscle strength prior to injury is not fully justified. There are indications that there might be a difference related to limb dominance [14,15]. The concept of limb dominance in the lower extremity, however, is controversial. Van der Harst et al. in a study of leg kinematics and kinetics in landing from a single-leg hop [16] defined the dominant leg as the leg with the biggest horizontal hop distance. Most investigators, on the other hand, seem to consider the leg preferred for kicking a ball as the dominant leg. If the leg preferred for kicking, which for the majority of people is the right leg [17], is defined as dominant, the contralateral leg would serve as supporting and weight bearing and therefore being stronger and heavier [15,18]. Considering the different views of leg dominance, it is not surprising that there is no consensus regarding difference in muscle cross sectional area (CSA) or volume comparing dominant and non-dominant leg.

The reduction of maximal voluntary contraction force (MVC) of quadriceps in the ACL-deficient knee is not only due to atrophy but also to arthrogenic muscle inhibition [19] with the effect of reduced maximal voluntary muscular activation (MVA) and it has been shown that this reduction is bilateral [8]. Berchuck et al. [20] studied gait adaptation in ACL-injured patients. They found that the subjects adapted their gait to reduce contraction of quadriceps and that they tended to walk with symmetrical gait which meant that the contralateral leg was almost equally affected. Thus even a unilateral injury will have effects on the contralateral leg which makes using the contralateral leg as comparison after ACL-injuries problematic.

Asymmetry due to limb dominance implies that the effect of ACL-injury might be different in right-sided and left-sided injuries. Moreover, there is growing evidence that gender differences in muscular strength and activation impact the risk of attaining an ACL-injury and therefore aspects of gender are important when evaluating the effect of an injury [21].

### Purpose of study

The purpose of this study is two-folded. First we wanted to study muscle atrophy after the acute stage, here defined as two months. We wanted to investigate if there were any correlations between the degree of atrophy and concomitant meniscal injury, time from injury or the subjects' physical activity level after injury.

Atrophy is generally assessed by comparing the injured leg with the contralateral. But we hypothesized that other factors could influence the difference between injured and non-injured leg, Therefore the second purpose was to investigate if other factors e g side of injury, effects of limb dominance or gender affected differences in muscle CSA. As no examinations were performed before the injury, the results are merely indirect and in this respect this paper serves more as a proof of concept.

## Methods

As part of a study of rehabilitation after surgical restoration of ACL-injuries, CSA of thigh muscles were obtained with computed tomography. The CT-scanning was performed just before surgery to serve as comparison for evaluating the effect on muscle size which was monitored during rehabilitation with repeated CT-scanning. Sixty-two ACL-deficient patients, 40 men and 22 women, were recruited and included when they were scheduled to undergo ACL reconstruction. Mean age with standard deviation was 28 ± 9 years for men (range 16–54) and 26 ± 8 years for women (range 16–45). Mean Body Mass Index (BMI) with standard deviation was 25.9 ±3.3 (range 19.8 – 32.4) for men and 24.4 ± 3.7 (range 18.9 – 31.8) for women. The most commonly reported activities before injury were soccer and alpine skiing but not all were active in sports. Of the 62 patients 26 had also sustained a meniscal tear diagnosed during a preceding arthroscopy or when surgery for ACL reconstruction was performed. CT-examinations were performed before surgery and time between injury and CT-examination ranged from 2 to 180 months with a median of 10 months. Three patients were excluded, one because of spontaneously gaining adequate stability in the knee due to the ACL scarring onto the posterior cruciate ligament, one because of cartilage injury in the joint and one because of unrelated symptoms from the contralateral knee. The Ethics Committee at the Karolinska Institutet approved the design of the study and the patients gave their informed consent for the planned procedures.

Of the 62 patients 36 (58%) had a left-side injury and 26 (42%) had a right-side injury. There were no differences between men and women, chi-square test for independence of gender and side was 0.68. Leg dominance, defined by the leg preferred for kicking a ball, was recorded for 46 patients where 43 had a right-sided dominance. Activity level before and after the ACL-injury was recorded according to the Tegner activity scale [22] and was also determined as stated in the International Knee Documentation Committee (IKDC) subjective knee evaluation form [23] on a four-graded scale from very strenuous, strenuous, moderate, to light.

### CT-imaging

Axial CT images with the patients in supine position were acquired 150 mm above the knee joint. Due to change of equipment at our department 4 different CT-scanners were used, although 53 of the 62 examinations were performed with a Philips Tomoscan SR 7000 (single slice helical CT-scanner, 100 kV and 75 mAs). Slice thickness in all images was 10 mm. The images were saved as DICOM-images in a PACS-system for later analysis.

The muscles analyzed were: quadriceps, sartorius, gracilis, semimembranosus, semitendinosus and biceps femoris. The different parts of quadriceps were not measured separately as especially vastus intermedius could not always be confidently separated from vastus lateralis and vastus medialis at this level. For the same reason the different parts of biceps femoris were not separated. The images were analyzed using NIH ImageJ version 1.42e software (http://rsbweb.nih.gov/ij/). The muscles were measured by outlining the borders and then registering the area with attenuation between 1 and 101 Hounsfield units. All measurements were made by one of the authors (SS) with 10 years of experience of CT-examinations. The method was previously evaluated with repeated measurements by two independent investigators and was found to have good reliability [24].

### Measurement evaluation

To evaluate the effect of injury, the percentage difference between the CSA of the injured and non-injured side was compared. The percentage difference in CSA was calculated for each muscle and for each patient separately. The mean of the percentage differences was then compared for all muscles for all patients together and also for right-sided and left-sided injuries separately. To test for side asymmetry the mean CSA for the muscles of the non-injured side in patients with right-sided injury was compared with those of the patients with left-sided injury for men and women separately. In the same way the mean CSA for muscles of the injured side was compared. As the measurements here refer to different individuals, absolute values of CSA in mm^2^ were used for comparison.

To test if there was any gender effect, the difference in injured and non-injured side CSA in per cent was compared between men and women. In the same way the potential effect of an additional meniscal tear was evaluated by comparing the percentage difference between injured and non-injured side in patients with meniscal tear with those without concomitant meniscal injury.

The CT-examinations were performed after the acute stage of the injury, here defined as two months. Although most of the patients were examined a rather long time after the injury, any remaining effect of time between injury and CT-examination was tested by classifying the patients in three groups, less than 6 months (N=17), 6 to 12 months (N=18) and more than 12 months (N=27) and then comparing the percentage difference in CSA. The classification was chosen in order to get reasonable sample size. In the same way correlation between degree of physical activity after the injury and the change in CSA was tested for patients with different level of activity.

### Statistical methods and data management

Repeated measurements analysis was used to analyze time dependent data and multiple comparisons of continuous data were performed by analysis of variance (ANOVA). The post-hoc procedure proposed by Fisher was used to control for multiplicity. Statistical comparisons in order to test differences between two independent groups were made by use of Student’s t-test for uncorrelated means. Within group analysis was made by use of pair-wise Student’s t-test for correlated means. In order to evaluate hypotheses of variables in contingency tables, chi-square test was used, or, in case of small expected frequencies, Fisher's Exact Test. The study employs multiple hypothesis testing, where each hypothesis was analyzed separately and the existence of patterns in results and the consistency of the results were considered in the analysis [25,26]. In addition, descriptive statistics was used to characterize the data. Data were expressed as mean ± SD. All analyses were carried out by use of a statistical software (The SAS system for Windows 9.2. SAS Institute Inc., Cary, NC, USA). A p-value of <0.05 was considered significant and in the case of a statistically significant result the probability value (p-value) was given.

## Results

### Injured versus non-injured side

Table 1 presents the CSA for the individual muscles comparing the injured with the non-injured side. A statistically significant smaller CSA of the injured side was found for the quadriceps muscle both when comparing all the patients and when comparing right-sided and left-sided injuries separately. On the other hand, with a right-sided injury, the CSA of all muscles except quadriceps tended to be larger on the injured side although the difference was statistically significant only for gracilis, semitendinosus and biceps femoris. With a left-sided injury the CSA of all the muscles on the injured side except semitendinosus tended to be smaller although the difference was statistically significant for quadriceps and semimembranosus only. The result for semitendinosus was highly influenced by two patients with left-sided injury who for some unknown reason had very small CSA of semitendinosus in the non-injured leg.

**Table 1:** **CSA in mm**^**2**^**, mean and SD, for injured and non-injured side respectively and mean of percentage difference between injured and non-injured side with p-value**

	**Injured**	**Non-injured**	**Diff%**	**p-value**
***All patients (N=62)***				
Quadriceps	5458 (1230)	5780 (1219)	−5.5%	<0.001
Sartorius	388 (103)	388 (105)	0.7%	0.583
Gracilis	337 (86)	328 (93)	4.3%	0.029
Semimembranosus	1331 (278)	1352 (266)	−1.2%	0.289
Semitendinosus	616 (237)	608 (231)	5.3%	0.134
Biceps femoris	1692 (355)	1673 (337)	1.5%	0.233
***Right-sided injury (N=26)***				
Quadriceps	5142 (1257)	5603 (1224)	−8.5%	< 0.001
Sartorius	393 (108)	382 (108)	3.7%	0.113
Gracilis	343 (94)	315 (97)	10.7%	0.005
Semimembranosus	1307 (302)	1283 (246)	1.7%	0.405
Semitendinosus	638 (253)	595 (221)	8.3%	0.024
Biceps femoris	1664 (402)	1595 (369)	4.4%	0.029
***Left-sided injury (N=36)***				
Quadriceps	5686 (1176)	5907 (1217)	−3.3%	0.024
Sartorius	385 (101)	393 (104)	−1.4%	0.371
Gracilis	332 (81)	337 (90)	−0.3%	0.870
Semimembranosus	1349 (262)	1401 (273)	−3.4%	0.013
Semitendinosus	600 (226)	617 (241)	3.1%	0.570
Biceps femoris	1712 (321)	1729 (305)	−0.6%	0.732

### Right versus left side

Table 2 presents mean CSA for the measured muscles for men and women separately for the leg without ACL-injury and similar figures for the injured leg. A comparison was made between right and left side i.e. the right side of patients with left-sided injury was compared with the left side of those with a right-sided injury. The number of patients in each group was low and there was a great variability as can be seen from standard deviation figures. None of the differences for the non-injured legs were statistically significant, but for 10 out of 12 comparisons the recorded mean CSA was larger for the right leg than for the left even though the difference for gracilis in women was marginal. For the ACL-deficient leg, CSA for quadriceps in women was smaller in right injured legs compared to left injured legs (p<0.05). There was also a difference in the effect of ACL-injury when comparing left-sided and right-sided injuries. This is illustrated in Table 3 which shows some of the figures from Table 2 reorganized to illustrate this. The table shows that the difference between injured and non-injured side was bigger for right-sided than for left-sided injuries. The mean percentage differences for quadriceps CSA between injured and non-injured leg for men and women combined were −8.5% and −3.3% for right-sided and left-sided injuries respectively (p=0.018). When separating for gender the corresponding figures were, -7.1% and −4.1% (p=0.294) for men and −10.7% and −1.7% (p=0.012) for women.

**Table 2:** **CSA in mm**^**2**^**, mean and SD, comparing right leg for patients with left-sided injuries with left leg for patients with right-sided injures and vice versa**

	**Right**	**Left**	**Diff**	**p-value**
***Leg without ACL-injury***				
***Men***				
Quadriceps	6504 (997)	6294 (982)	210	0.517
Sartorius	446 (81)	429 (107)	17	0.563
Gracilis	364 (93)	337 (68)	27	0.321
Semimembranosus	1495 (247)	1382 (255)	112	0.172
Semitendinosus	611 (277)	666 (209)	−56	0.500
Biceps femoris	1845 (256)	1750 (361)	95	0.337
***Women***				
Quadriceps	4714 (553)	4497 (588)	218	0.382
Sartorius	285 (42)	306 (53)	−21	0.307
Gracilis	283 (55)	281 (128)	2	0.958
Semimembranosus	1215 (231)	1124 (116)	91	0.272
Semitendinosus	629 (154)	482 (198)	147	0.065
Biceps femoris	1497 (266)	1347 (224)	149	0.175
***ACL-deficient leg***				
***Men***				
Quadriceps	5847 (1002)	6219 (1055)	−371	0.273
Sartorius	445 (105)	434 (82)	12	0.693
Gracilis	369 (70)	355 (83)	14	0.576
Semimembranosus	1457 (286)	1449 (239)	8	0.924
Semitendinosus	731 (247)	609 (252)	122	0.138
Biceps femoris	1858 (361)	1838 (288)	19	0.854
***Women***				
Quadriceps	4012 (643)	4620 (463)	−608	0.018
Sartorius	309 (35)	288 (53)	22	0.277
Gracilis	301 (114)	286 (56)	15	0.701
Semimembranosus	1068 (115)	1149 (183)	−81	0.240
Semitendinosus	489 (189)	582 (171)	−93	0.241
Biceps femoris	1356 (243)	1460 (223)	−105	0.305

Differences between right and left side with p-value.

**Table 3:** **Mean CSA in mm**^**2 **^**for quadriceps of injured and non-injured leg for left sided and right sided injuries separately for men and women respectively**

	**Men (N=40)**			**Women (N= 22)**		
	**Injured**	**Non-injured**	**Diff.**	**Injured**	**Non-injured**	**Diff.**
**Left**	6218	6294	−76	4620	4497	124
**Right**	5847	6504	−656	4012	4714	−702
**Difference**	−371	209		−608	218	

### Gender

As expected, there was a difference in CSA in general between men and women both for the injured and non-injured leg, which is shown in Table 2. Table 4 shows the difference in CSA between injured and non-injured leg in per cent with a comparison between men and women. The only statistically significant difference was for semimembranosus where the reduction of CSA was bigger in women than in men. The tendency was the same when calculating right-sided and left-sided injuries separately, although the difference was statistically significant for right-sided injuries only.

**Table 4:** **Mean CSA in mm**^**2**^**for muscles of injured and non-injured leg with percentage difference and a comparison between men and women**

	**Men (N=40)**			**Women (N= 22)**			
	**Injured**	**Non-injured**	**Diff%**	**Injured**	**Non-injured**	**Diff%**	**p-value**
Quadriceps	6070	6420	−5.3%	4344	4615	−5.8%	0.833
Sartorius	438	440	0.2%	298	294	1.7%	0.613
Gracilis	361	353	3.7%	293	282	5.4%	0.679
Semimembranosus	1452	1450	0.7%	1112	1174	−4.7%	0.026
Semitendinosus	657	633	9.5%	540	562	−2.3%	0.108
Biceps femoris	1846	1807	2.6%	1413	1429	−0.4%	0.256

### Meniscal injury

Table 5 shows the difference in CSA between the injured and the non-injured leg for patients with or without meniscal tear. No significant differences were found except for semitendinosus but the figures for semitendinosus were highly influenced by the two patients mentioned above, both with left-sided injury and meniscal tear.

**Table 5:** **Mean CSA in mm**^**2 **^**for muscles of injured and non-injured leg with percentage difference and a comparison between patients with and without meniscal tear**

	**Meniscal tear (N=26)**			**No meniscal tear (N= 36)**			
	**Injured**	**Non-injured**	**Diff%**	**Injured**	**Non-injured**	**Diff%**	**p-value**
Quadriceps	5631	5876	−4.1%	5332	5710	−6.5%	0.292
Sartorius	411	408	0.5%	372	373	0.9%	0.872
Gracilis	361	349	4.9%	319	313	3.9%	0.797
Semimembranosus	1369	1380	−0.7%	1304	1331	−1.6%	0.713
Semitendinosus	644	595	14.9%	595	617	−1.6%	0.018
Biceps femoris	1714	1708	0.6%	1677	1647	2.2%	0.546

### Time from injury and physical activity

No significant differences were found when comparing patients with different time between injury and CT-examination. Activity level according to the Tegner scale before and after injury was 7.8 ± 1.0 and 3.1 ± 1.6 respectively for men and 7.0 ±1.4 and 2.4 ± 1.1 for women. The self-estimated physical activity before and after the injury is presented in Table 6. The differences between the injured and non-injured side were the same irrespective of activity after injury although there was a tendency to less difference in the two patients who retained very strenuous activity.

**Table 6:** Self-estimated physical activity

	**Very strenuous**	**Strenuous**	**Moderate**	**Light**
**Before injury**	52	8	2	0
**After injury**	2	8	36	16

## Discussion

In agreement with our hypothesis we found that apart from the ACL-injury there were also other factors that influenced the comparison between injured and non-injured muscle CSA. We found several differences between the left and right leg. The CSA of the muscles of the right leg, which for the majority of the patients was the dominant leg, was bigger than the CSA of the left leg. There was also a side difference in the effect of ACL-injury since the difference in CSA of quadriceps was more pronounced after a right-sided injury than after a left-sided injury.

It is previously known that the most obvious effect of ACL-injury on muscle size is atrophy of quadriceps. Williams et al. [4] found 4.5-13% lower muscle volume of quadriceps in the injured knee compared to the opposite side for patients defined as “non-copers” i.e. patients who do not compensate well for the injury. In our study the CSA of quadriceps was approximately 5% smaller in the affected side which is in agreement with these findings. Usually the contralateral leg is used as control as it provides a matched sample which eliminates the high interindividual variability of the muscles due to different statue and physical activity. As pointed out in the introduction, this ignores any difference between the sides of a healthy person and also the effect an injury has on the contralateral leg. It has also been demonstrated that there are side differences in other anatomical characteristics in the lower extremity as e g Q-angle and tibial torsion [27]. In this study we tried to check the feasibility of using the contralateral leg for comparison. The results are mainly indirect as we did not have an examination before the injury. In literature there are conflicting results as to which leg is the heaviest with the greatest muscle mass. There are also indications that persons who suffer ACL-injuries are not representative in this respect as imbalance between quadriceps and hamstrings might influence the risk of attaining an ACL-injury [28].

The new findings in this study are the differences between left and right leg. Generally the CSA of the right leg, which for the majority of the patients was the dominant leg defined as the leg preferred for kicking a ball, was bigger than the CSA of the left leg. Even more interesting is that there was a side difference in the effect of ACL-injury since the difference in CSA of quadriceps between the injured and non-injured side was more pronounced after a right-sided injury than after a left-sided injury. There was also a gender difference as reduction of CSA of semimembranosus was more pronounced in females.

### Right versus left side

As our population already had an ACL-injury, we had to analyze side differences in the non-injured leg by comparing the right leg in patients with a left-sided injury with the left leg in patients with right-sided injury. The high inter individual variability shown by the values of standard deviation in Table 2 made the statistical power of such a comparison low and we did not find any statistically significant differences. Nevertheless, in 10 out of 12 comparisons the recorded mean CSA of the right leg was larger than that of the left leg. The probability for such a skewed distribution if there is no side difference is 0,039 (double sided binomial distribution). Further support for the view that the muscles of the right leg were bigger than those of the left leg came from comparing the matched samples of injured versus non-injured side separated for left-sided and right-sided injuries in Table 1. Quadriceps was smaller on the injured side, probably due to atrophy as expected. Except for quadriceps, the CSA of the other muscles of the injured side tended to be bigger than on the non-injured side when the right leg was injured. The opposite was true for left-sided leg injury where the muscular CSA in general was smaller in the non-injured leg.

The other observed side asymmetry in this study was the effect of ACL-injury on muscle size. Table 3 shows that the difference between the injured and the non-injured side in quadriceps CSA was larger for right-sided than for left-sided injuries which implies that the atrophy of quadriceps was more pronounced in right-sided injuries. Even if the reason for this is not clear, it is likely to be related to limb dominance and maybe also to gender differences as the effect seemed to be greater in women than in men.

The present study thus indicates that there are differences in muscle size both between right and left side in general and in the effect of an ACL-injury that have to be taken in account when evaluating atrophy after ACL injury.

### Dominance

Although leg dominance was not determined for all patients included in this study, a great majority of the patients were right-leg dominant for kicking. This is in accordance with previous findings where e.g. Matava et al. reported a figure of 89% [17]. Our findings strongly suggest that for our study population the muscles of the right thigh, which for the majority is the leg preferred for kicking, had larger CSA, than the muscles of the left thigh. These findings are however indirect as they are based on comparison of the muscles of the right thigh in patients with a left-sided injury with the left thigh in patients with a right-sided injury.

In a study of skeletons Čuk et al. [14] found that the left femur was larger and longer than the right which was interpreted as the left limb being the supportive leg for both left-handers and right-handers. This would indicate that the left leg would be stronger and heavier which was also found for most of the subjects in a cadaver study of Chhibber [15]. Peters [18] in a review-article concluded that in adult right-handers the left leg is longer and heavier than the right leg. This finding was however not confirmed in a study of Tate et al. [29] where differences in vastus lateralis and medialis were found between the dominant and non-dominant leg but otherwise there were no differences in total quadriceps and only small differences in the other muscles. In a study of age-associated strength change in women [30] the subject of limb dominance was also analyzed. Limb dominance was defined as the preferred limb and CSA of thigh lean mass was calculated from anthropometric measurements. They reported a significant larger CSA in the dominant limb. There are thus conflicting results regarding the differences in muscle size between sides that might be at least partly due to different definition of dominance.

### Gender

Women’s semimembranosus CSA was significantly reduced in the injured leg which was not the case for men. From our data it is not possible to deduce if this was due to more pronounced atrophy after the injury or whether a smaller semimembranosus might have affected the risk of getting an ACL-injury. But it has been demonstrated that in female athletes who subsequently suffered ACL-injuries there was strength imbalance between the quadriceps and hamstring muscle groups that was not found among men [28] and this supports the view that a small semimembranosus might be an etiologic factor for ACL-injuries. Gender differences in muscular activation of quadriceps and hamstring have also been reported by Chappel et al. [31].

In our study there was no gender difference between the proportions of left-sided versus right-sided injuries. This means that we could not confirm the findings of other studies where e.g. Negrete et al. reported an increased proportion of left-sided non contact ACL-injuries in females [32]. Brophy et al. [21] found gender differences in the frequency of non-contact ACL injuries in soccer players where females were more likely to injure their supporting leg whereas males tend to injure their kicking leg. This was also found in a recent study of recreational skiers where 90% had a right-sided lower limb dominance defined as the leg preferred for kicking a ball. Sixty-eight per cent of the females injured their left leg versus 48% of the males [33].

### Meniscal injury

The present study did not find any difference in muscle CSA of ACL-deficient patients with or without meniscal tear. This is in agreement with previous literature. Akima et al. [13] showed that there was atrophy of quadriceps after meniscal lesions and partial arthroscopic meniscectomy whereas the other muscle groups were not affected. Urbach et al. [8] found that the MVC was more reduced in patients with a combined ACL and additional knee injuries compared with patients with isolated ACL-injury but concluded that this was an effect of decreased MVA alone and not due to any differences in atrophy.

### Physical activity and time from injury

Most of the patients in the present study had reduced their physical activity considerably as shown by the reduction of Tegner activity score after injury and IKDC score (Tab 6), and all were awaiting ACL-reconstruction due to problems with knee instability. They could therefore be defined as non-copers. There is no accepted definition for the acute stage of an ACL-injury which can range from days to weeks [10] but as the subjects in this study were examined at least 2 months after the injury and with a median time of 10 months, they are here considered as after the acute stage. Williams et al. [4] studied quadriceps weakness and atrophy in predicted non-copers with an average time of two months after injury and found significant reduction in volume and CSA of quadriceps. The atrophy of quadriceps thus seems to appear rather early and to persist in untreated chronic ACL-deficient patients [3]. The patients in our study were non-copers who had reduced their physical activity a great deal, and the CT-examination was performed after the acute stage. It is thus not surprising that we could not find any correlation between the degree of reduced physical activity or the time from injury and the difference in CSA.

### Limitations

The observed differences between right and left side which are part of the important findings in this study are based on comparing groups of patients instead of comparing right and left side in the same individual as we had no CT-examinations before the ACL-injury. This results in statistically less powerful comparisons. An alternative is to examine a group of healthy individuals but as imbalance in the muscles of the thigh might be an etiologic factor for attaining ACL-rupture, such a study group might not be representative for patients with ACL-rupture.

Another weakness of the study is that muscle size is estimated from measuring CSA instead of muscle volume. This was because we, in attempt to keep the radiation dose low for this study group who voluntarily participated in the study, decided to use only discrete axial CT-images at certain levels instead of a complete examination of the thigh. An alternative would have been to use magnetic resonance imaging (MRI) instead of CT but even with MRI measuring volumes of the entire muscle is difficult and time consuming as different parts of the muscles are not always readily separable.

## Conclusions

The contralateral leg is often the used for evaluating the effects of ACL-injury and for monitoring the result of rehabilitation, but this study indicates that there is asymmetry in the muscles that ought to be taken in account and, that the effect of an ACL-injury on muscle size differs between right-sided and left-sided injuries. The results need to be confirmed in future studies. There is also growing evidence that dominance- and gender-effects, such as our finding of decreased female semimembranosus CSA, have to be considered when assessing the effect of ACL-injury and the result of rehabilitation.

## Competing interests

The authors declare that they have no competing interests.

## Authors' contributions

SS carried out measurements of the CT images, participated in statistical analysis and drafted the manuscript. ML recorded activity scores and participated in drafting of the manuscript. MLW participated in the design of the study, coordination of the examinations and drafting of the manuscript. PA participated in interpretation and analysis of the data and in drafting of the manuscript. AS participated in the design of the study, coordination of the examinations and in drafting of the manuscript. All authors read and approved the final manuscript.

## Pre-publication history

The pre-publication history for this paper can be accessed here:

http://www.biomedcentral.com/1471-2474/14/150/prepub

## References

[B1] Baugher WH Warren RF Marshall JL Joseph A Quadriceps atrophy in the anterior cruciate insufficient knee Am J Sports Med 1984 12 3 192 195 10.1177/036354658401200304 6742300

[B2] Gerber C Hoppeler H Claassen H Robotti G Zehnder R Jakob RP The lower-extremity musculature in chronic symptomatic instability of the anterior cruciate ligament J Bone Joint Surg Am 1985 67 7 1034 1043 4030823

[B3] Lorentzon R Elmqvist LG Sjostrom M Fagerlund M Fuglmeyer AR Thigh musculature in relation to chronic anterior cruciate ligament tear: muscle size, morphology, and mechanical output before reconstruction Am J Sports Med 1989 17 3 423 429 10.1177/036354658901700318 2729494

[B4] Williams GN Snyder-Mackler L Barrance PJ Buchanan TS Quadriceps femoris muscle morphology and function after ACL injury: a differential response in copers versus non-copers J Biomech 2005 38 4 685 693 10.1016/j.jbiomech.2004.04.004 15713288

[B5] Elmqvist LG Lorentzon R Johansson C Fugl-Meyer AR Does a torn anterior cruciate ligament lead to change in the central nervous drive of the knee extensors? Eur J Appl Physiol Occup Physiol 1988 58 1–2 203 207 3203669 10.1007/BF00636627

[B6] Snyder-Mackler L De Luca PF Williams PR Eastlack ME Bartolozzi AR 3rd Reflex inhibition of the quadriceps femoris muscle after injury or reconstruction of the anterior cruciate ligament J Bone Joint Surg Am 1994 76 4 555 560 8150823 10.2106/00004623-199404000-00010

[B7] Hurley MV The effects of joint damage on muscle function, proprioception and rehabilitation Man Ther 1997 2 1 11 17 10.1054/math.1997.0281 11440520

[B8] Urbach D Nebelung W Ropke M Becker R Awiszus F Bilateral dysfunction of the quadriceps muscle after unilateral cruciate ligament rupture with concomitant injury central activation deficit Unfallchirurg 2000 103 11 949 955 10.1007/s001130050651 11142883

[B9] Ingersoll CD Grindstaff TL Pietrosimone BG Hart JM Neuromuscular consequences of anterior cruciate ligament injury Clin Sports Med 2008 27 3 383 404 vii 10.1016/j.csm.2008.03.004 18503874

[B10] Beynnon BD Johnson RJ Abate JA Fleming BC Nichols CE Treatment of anterior cruciate ligament injuries, part I Am J Sports Med 2005 33 10 1579 1602 10.1177/0363546505279913 16199611

[B11] Beynnon BD Johnson RJ Abate JA Fleming BC Nichols CE Treatment of anterior cruciate ligament injuries, part 2 Am J Sports Med 2005 33 11 1751 1767 10.1177/0363546505279922 16230470

[B12] Hurd WA M Snyder Mackler L Management of the Athlete With Acute Anterior Cruciate Ligament Deficiency Sports Health Multidisciplinary Approach 2009 1 39 46 10.1177/1941738108326977 PMC3445111 23015853

[B13] Akima H Furukawa T Atrophy of thigh muscles after meniscal lesions and arthroscopic partial menisectomy Knee Surg Sports Traumatol Arthrosc 2005 13 8 632 637 10.1007/s00167-004-0602-9 15827765

[B14] Cuk TLSP Stefancic M Lateral Asymmetry of Human Long Bones Variability Evol 2001 9 19 32

[B15] Chhibber SR Singh I Asymmetry in muscle weight and one-sided dominance in the human lower limbs J Anat 1970 106 Pt 3 553 556 5423943 PMC1233429

[B16] Van der Harst JJ Gokeler A Hof AL Leg kinematics and kinetics in landing from a single-leg hop for distance. A comparison between dominant and non-dominant leg Clin Biomech (Bristol, Avon) 2007 22 6 674 680 10.1016/j.clinbiomech.2007.02.007 17418922

[B17] Matava MJ Freehill AK Grutzner S Shannon W Limb dominance as a potential etiologic factor in noncontact anterior cruciate ligament tears J Knee Surg 2002 15 1 11 16 11829328

[B18] Peters M Footedness: asymmetries in foot preference and skill and neuropsychological assessment of foot movement Psychol Bull 1988 103 2 179 192 3283813 10.1037/0033-2909.103.2.179

[B19] Palmieri-Smith RM Thomas AC Wojtys EM Maximizing quadriceps strength after ACL reconstruction Clin Sports Med 2008 27 3 405 424 vii-ix 10.1016/j.csm.2008.02.001 18503875

[B20] Berchuck M Andriacchi TP Bach BR Reider B Gait adaptations by patients who have a deficient anterior cruciate ligament J Bone Joint Surg Am 1990 72 6 871 877 2365720

[B21] Brophy R Silvers HJ Gonzales T Mandelbaum BR Gender influences: the role of leg dominance in ACL injury among soccer players Br J Sports Med 2010 44 10 694 697 10.1136/bjsm.2008.051243 20542974

[B22] Tegner Y Lysholm J Rating systems in the evaluation of knee ligament injuries Clin Orthop Relat Res 1985 198 43 49 4028566

[B23] Irrgang JJ Anderson AF Boland AL Harner CD Kurosaka M Neyret P Richmond JC Shelborne KD Development and validation of the international knee documentation committee subjective knee form Am J Sports Med 2001 29 5 600 613 11573919 10.1177/03635465010290051301

[B24] Strandberg S Wretling ML Wredmark T Shalabi A Reliability of computed tomography measurements in assessment of thigh muscle cross-sectional area and attenuation BMC Med Imagin 2010 10 08 10.1186/1471-2342-10-8 PMC2928755 20701775

[B25] Montgomery DC Design and Analysis of Experiments, 3 ed edn 1991 New York: John Wiley & Sons

[B26] Daniel WW Biostatistics: A Foundation for Analysis in the Health Sciences, 6th ed edn 1995 New York: John Wiley & Sons

[B27] Shultz SJ Nguyen AD Bilateral Asymmetries in clinical measures of lower-extremity Anatomic characteristics Clin J Sport Med 2007 17 5 357 361 10.1097/JSM.0b013e31811df950 17873547

[B28] Myer GD Ford KR Barber Foss KD Liu C Nick TG Hewett TE The relationship of hamstrings and quadriceps strength to anterior cruciate ligament injury in female athletes Clin J Sport Med 2009 19 1 3 8 10.1097/JSM.0b013e318190bddb 19124976 PMC9928500

[B29] Tate CM Williams GN Barrance PJ Buchanan TS Lower extremity muscle morphology in young athletes: an MRI-based analysis Med Sci Sports Exerc 2006 38 1 122 128 10.1249/01.mss.0000179400.67734.01 16394964

[B30] Hunter SK Thompson MW Adams RD Relationships among age-associated strength changes and physical activity level, limb dominance, and muscle group in women J Gerontol A Biol Sci Med Sci 2000 55 6 B264 273 10.1093/gerona/55.6.B264 10843342

[B31] Chappell JD Creighton RA Giuliani C Yu B Garrett WE Kinematics and electromyography of landing preparation in vertical stop-jump: risks for noncontact anterior cruciate ligament injury Am J Sports Med 2007 35 2 235 241 17092926 10.1177/0363546506294077

[B32] Negrete RJ Schick EA Cooper JP Lower-limb dominance as a possible etiologic factor in noncontact anterior cruciate ligament tears J Strength Cond Res 2007 21 1 270 273 10.1519/00124278-200702000-00048 17313288

[B33] Ruedl G Webhofer M Helle K Strobl M Schranz A Fink C Gatterer H Burtscher M Leg dominance is a risk factor for noncontact anterior cruciate ligament injuries in female recreational skiers Am J Sports Med 2012 AJSM PreView, published on March 16, 2012 as 10.1177/0363546512439027 22427619

